# Beneficial Impact of Kaempferol on Kidney Function and Long‐Term Prognosis in Overweight or Obese Adults

**DOI:** 10.1002/fsn3.71393

**Published:** 2026-01-27

**Authors:** Lin Shi, Yiquan Sang

**Affiliations:** ^1^ Department of Gastroenterology Xuzhou Central Hospital, Southeast University Xuzhou China; ^2^ Beijing Tsinghua Changgung Hospital Beijing China; ^3^ Department of Endocrinology Xuzhou Central Hospital, Southeast University Xuzhou China

**Keywords:** dietary kaempferol, kidney function, long term prognosis, obesity

## Abstract

Obesity is a significant risk factor for chronic kidney disease (CKD). Kaempferol, a natural flavonoid, has the ability to alleviate oxidative stress in animal models. The aim of this study is to assess the relationship between kaempferol intake and renal function, as well as its impact on long‐term prognosis in the obese population. This is an observational, cross‐sectional, and longitudinal analysis based on NHANES data (2007–2018). Data regarding flavanol consumption were obtained from FNDDS. Prognostic information was sourced from the NCHS. Multivariate logistic and Cox regression, subgroup, and sensitivity analysis were used to investigate the relationship between dietary kaempferol and kidney function and prognosis. A total of 9816 participants with a median follow‐up of 117 months were included. The stratified analysis revealed that kaempferol was a protective factor for renal function. For every 5 mg/day increment in kaempferol intake, the prevalence of kidney damage declined by 7% [OR = 0.93, 95% CI: 0.88–0.99]. Additionally, at a median follow‐up duration of 117 months, for every 5 mg/day increase in kaempferol intake, the mortality rate decreased by 7% [HR = 0.93, 95% CI: 0.85–0.98]. Our findings suggested that higher kaempferol intake is associated with a reduced risk of kidney damage and improved long‐term prognosis in obese individuals.

## Graphical abstract

Higher kaempferol intake is associated with reduced kidney damage prevalence and improved long‐term survival in overweight or obese adults. Each 5 mg/day increase in dietary kaempferol correlates with a 7% lower risk of kidney impairment and a 7% decrease in mortality over a median 117‐month follow‐up. These findings highlight kaempferol's potential renal and prognostic benefits in this population.

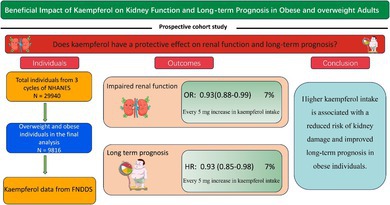

## 1 Introduction

Obesity or overweight has emerged as a major public health challenge, affecting approximately 12% of the world's adult population (Yau et al. 2024). Obesity is a well‐established risk factor for CKD, with studies showing that individuals with obesity are at a 3.6 times higher risk of developing CKD compared to those with normal weight (Hsu et al. 2006). The worldwide prevalence of CKD is estimated to affect more than 800 million individuals, and obesity has been identified as a major driver of this increasing trend (Kovesdy 2022).

The underlying mechanisms linking obesity to CKD are multifaceted, involving metabolic syndrome, hypertension, insulin resistance, and chronic inflammation, all of which contribute to progressive kidney damage, whereas reverse causality (CKD leading to obesity) is less commonly supported by evidence (Yau et al. 2024; Kovesdy 2022). Current therapeutic strategies for managing obesity‐related CKD, such as lifestyle interventions, antihypertensive medications, and antidiabetic treatments, are often inadequate to fully halt disease progression (Apperloo et al. 2024). Given the complex pathophysiology of obesity‐related kidney damage, alternative and adjunctive therapeutic approaches are urgently needed.

In recent years, natural compounds have garnered increasing attention for their potential role in preventing metabolic diseases and related organ damage (Alkandahri et al. 2024; Chaubey and Singh 2024). Kaempferol, a flavonoid with established antioxidant and anti‐inflammatory properties, plays a significant role in renal function and prognosis (Alkandahri et al. 2024; Shi et al. 2025). Predictively, kaempferol's ability to modulate oxidative stress and inflammation signifies its potential in early detection of renal impairment by mitigating biomarkers associated with kidney damage (Alshehri 2023). Preventively, it shields the kidneys from toxic insults, such as those caused by chemotherapeutic agents, by activating cellular defense mechanisms, including the Nrf2/ARE and SIRT1 pathways, thereby preserving renal function (Wang et al. 2020; Luo et al. 2021). Certainly, preliminary clinical studies indicate that kaempferol‐rich interventions, such as specific diets or supplements, can reduce inflammatory biomarkers and improve metabolic parameters in patients with conditions like diabetic nephropathy (Shi et al. 2025; Hosseinpour‐Niazi et al. 2015; Noroozi et al. 2000). Kaempferol was selected for analysis because it has demonstrated potent antioxidative and anti‐inflammatory effects in preclinical models of kidney injury. However, population‐based data on the relationship between dietary kaempferol intake and kidney health are scarce. Although kaempferol itself is not yet approved by the U.S. Food and Drug Administration (FDA) as a therapeutic agent, its biological activities suggest potential clinical relevance and warrant further investigation in human populations.

Therefore, this study aimed to evaluate the association between dietary kaempferol intake and kidney function as well as long‐term prognosis in overweight or obese adults, using data from a nationally representative cohort. We hypothesized that higher dietary kaempferol intake, reflecting natural consumption through habitual diet, is associated with improved renal function and survival outcomes.

## 2 Materials and Methods

### 2.1 Study Population

The dataset for our analysis was extracted from three distinct cycles of the National Health and Nutrition Examination Survey (NHANES): 2007–2008, 2009–2010, and 2017–2018 (Zheng et al. 2024; Shi 2023). Because the data on flavonoids intake was only available in these three cycles, we chose the three cycles of 2007, 2009, and 2017. Our study population was individuals who were overweight or obese. We applied specific exclusion criteria, which included: (1) individuals who were pregnant, (2) those with incomplete records regarding kidney function or flavonoids intake, and (3) individuals whose body mass index (BMI) could not be calculated. Comprehensive details regarding the NHANES methodology and the procedures for data collection are documented in prior publications. Initially, participants were interviewed in their residences to collect demographic data, which was followed by an examination at a mobile examination facility for further data acquisition. The study's protocols were scrutinized and endorsed by the National Center for Health Statistics (NCHS) Ethics Review Board, and each participant provided written consent. The research was conducted in accordance with the ethical standards set forth in the Declaration of Helsinki. The datasets utilized in this study were publicly available and ensured confidentiality.

### 2.2 Assessment of Kidney Function and Prognosis

In this study, obesity was categorized as a BMI exceeding 30 kg/m^2^, and being overweight was defined as a BMI ranging from 25 to 30 kg/m^2^ in accordance with WHO (Hsu et al. 2006). We evaluated two primary indicators of kidney impairment: the urinary microalbumin to creatinine ratio (UACR) and the estimated glomerular filtration rate (eGFR). Kidney impairment was identified by an eGFR below 60 mL/min/1.73 m^2^ and/or a UACR exceeding 30 mg/g. The eGFR was determined using the CKD‐EPI formula, as recommended by the Kidney Disease: Improving Global Outcomes (KDIGO) guidelines (Kovesdy 2022; Kidney Disease: Improving Global Outcomes CKDWG 2024). Both UACR and eGFR assessments were performed at the mobile examination center. The study analyzed survival rates as an indicator of long‐term prognosis. Data on mortality and follow‐up were obtained from the 2019 Linked Mortality Files made publicly available by the NCHS, with follow‐up extending from the time of survey participation to December 31, 2019.

### 2.3 Estimation of Dietary Kaempferol

The data on kaempferol intake dosage in this study were sourced from the Food and Nutrient Database for Dietary Studies (FNDDS) flavonoid database. Consumption information for four flavonoids, Kaempferol, Theaflavin, Isorhamnetin, Luteolin, was reported separately. For each participant, daily intake values for these four flavanols on both day 1 and day 2 were recorded. Based on the FNDDS flavonoid database, the intake used in this study originated from the participants' daily diet. The What We Eat in America (WWEIA) NHANES dietary intake data were based on 24‐h recalls, collected using the USDA Automated Multiple‐Pass Method. The Flavonoid Intake Data Files allow estimation of flavonoid intake for the population and subgroups, linking intake data to other dietary and health‐related information collected in NHANES from 2007 to 2008, 2009–2010, and 2017–2018. The flavonoid database enables estimation of kaempferol intake dosage for our study participants and helps identify potential associations between kaempferol intake and health measures of interest. We selected flavonoid intake data reported for both day 1 and day 2, using the two‐day average in our analysis to reduce bias (Zheng et al. 2024).

### 2.4 Covariates

Data on demographics and laboratory parameters were extracted and consolidated from the NHANES database. The demographic variables included age, gender, ethnicity (White, Black, Mexican, and other), and the poverty‐to‐income ratio (PIR). Smoking status was categorized based on self‐reported information: never smokers (individuals who smoked fewer than 100 cigarettes in their lifetime and were not current smokers), former smokers (those who smoked more than 100 cigarettes in their lifetime but were not current smokers), and current smokers (those who smoked at least 100 cigarettes in their lifetime and were current smokers). Alcohol consumption was classified as follows: never (fewer than 12 lifetime drinks), former (12 or more lifetime drinks but none in the past year), current light/moderate (up to 1 drink per day on average for women or up to 2 drinks per day on average for men over the past year), and current heavier (more than 1 drink per day on average for women or more than 2 drinks per day on average for men over the past year) (Shi et al. 2023; Guo et al. 2024). Medical histories, including diabetes mellitus and hypertension, were determined through self‐reported data, laboratory tests, or imaging studies. Laboratory measurements included serum albumin, glycosylated hemoglobin (HbA1c), glucose, insulin, uric acid, and blood urea nitrogen. Detailed procedures for specimen collection, processing, quality assurance, and monitoring are described elsewhere (Shi et al. 2023; Guo et al. 2024).

### 2.5 Statistical Analyses

The statistical design and methods in this study follow the following several steps:

#### 2.5.1 Step 1: Comparison of Baseline Data

According to the renal function of the study participants, we divided the researchers into the normal renal function group and the renal function impairment group. Continuous variables were reported as mean ± standard deviation (*M* ± SD) and comparisons between groups were performed using either a *T*‐test or the Wilcoxon rank‐sum test, depending on the data distribution. Categorical variables were presented as percentages (%) and analyzed using the Chi‐square test or Fisher's exact test, as appropriate.

#### 2.5.2 Step 2: The Relationship Between Flavonoids and Renal Function in the Study Population

After grouping the participants, we compared the risk factors for kidney injury, such as age, gender, diabetes, hypertension, etc., according to the above comparison methods, and also compared several flavonoids (Kaempferol, Theaflavin, Isorhamnetin, Luteolin). Afterwards, in order to clarify the relationship between flavonoids and kidney injury, we conducted multivariate logistic regression after weight correction. During this process, we constructed multiple regression models (Model 1, Model 2, Model 3). We also compared the relationships between four different flavonoids (Kaempferol, Theaflavin, Isorhamnetin, Luteolin) and abnormal renal function in the multivariate regression model. The specific adjustment variables for each model can be seen in the footnote of each table. Multicollinearity was assessed using the variance inflation factor (VIF), and all values were below 5, indicating no serious multicollinearity.

#### 2.5.3 Step 3: The Association Between Renal Function, Survival Status and Kaempferol

In order to further explore the relationship between kaempferol and the prevalence of decreased renal function, we converted kaempferol intake from a continuous variable into a categorical one, based on tertiles (Tertile 1, Tertile 2, Tertile 3; T1, T2, T3). Using the lowest tertile (Tertile 1) as the reference, we performed multivariate logistic regression to evaluate the association between Kaempferol intake and the prevalence of kidney impairment among overweight or obese participants. The specific adjustment variables for each model are outlined in Table 3. In addition, we also used restricted cubic spline (RCS) analysis to investigate potential non‐linear associations between Kaempferol intake and the decline in kidney function. In the following analysis, we further explored the relationship between kaempferol and the prognosis of overweight or obese populations. Kaplan–Meier (KM) survival curve and Cox regression analysis were used to explore whether kaempferol had a protective effect on the long‐term prognosis of overweight or obese populations. We constructed multivariate Cox regression models (model1, model2, model3) to test whether kaempferol has a protective effect on the long‐term prognosis of overweight or obese individuals. The specific variables adjusted in each model can be seen in the footnote of Table 4. Similarly, as a sensitivity analysis, we also conducted the same analysis again after converting kaempferol intake into a categorical variable. Moreover, we also used RCS analysis to investigate potential non‐linear associations between kaempferol intake and the survival status. Afterwards, in order to clarify the effect of kaempferol on renal function and prognosis in different subgroup populations, we conducted subgroup analyses in different populations (gender, age, overweight or obesity, and diabetes status).

In the subsequent analysis, considering that there is no gold standard for the intake of Kaempferol, we adopted the approach from a previous study. We categorized the participants into the low‐intake and high‐intake groups based on the median intake of kaempferol for each participant. Furthermore, we explored the relationship between kaempferol and renal function as well as the prognosis of the study population.

#### 2.5.4 Step 4: Sensitivity Analysis

To verify the stability of the results, we conducted a sensitivity analysis. In the sensitivity analysis, we first grouped the data according to different cycles. Then, we grouped kaempferol again according to the median value following the methods in previous literature, dividing it into the low‐intake group and the high‐intake group. Then, we analyzed renal function and prognosis again within different cycles. All statistical analyses were performed by R software. A *p* < 0.05 was considered statistically significant.

## 3 Results

### 3.1 Basic Characteristics of the Study Population

The total number of people in the three cycles was 29,940. After excluding 11,509 participants who were younger than 18 years or pregnant, and another 8615 individuals with incomplete data on renal function, flavonoid intake, or BMI, 9816 participants were ultimately included in the final analysis. The participant selection process is illustrated in Figure S1. Based on renal function status, we categorized the overweight and obese population into two groups: the normal renal function group and the renal impairment group, as detailed in Table 1. Out of the 9816 participants, 1907 exhibited renal impairment. The group‐specific characteristics are outlined in Table 1. Compared to the normal renal function group, participants with renal impairment were older, had a higher proportion of females, and exhibited greater BMI and waist circumference (*p* < 0.0001). Additionally, this group demonstrated elevated levels of HbA1c, blood glucose, and insulin, uric acid, with a higher prevalence of DM and hypertension (*p* < 0.0001). Regarding flavonoid intake, participants with renal impairment had significantly lower intakes of theaflavin, isorhamnetin, luteolin, and kaempferol compared to those with normal renal function.

**TABLE 1:** Baseline characteristics of study population.

Variable	Total	No kidney damage	Kidney damage	*p*
Age, year	48.61 (0.32)	46.23 (0.33)	61.76 (0.45)	< 0.0001
Sex, %				
Female	4995 (49.39)	4012 (48.40)	983 (54.87)	< 0.001
Male	4821 (50.61)	3897 (51.60)	924 (45.13)	
Ethnicity, %				
Black	2052 (11.46)	1615 (11.19)	437 (13.00)	< 0.0001
Mexican	1798 (9.42)	1520 (9.69)	278 (7.98)	
White	4291 (67.46)	3362 (66.95)	929 (70.27)	
Other	1675 (11.65)	1412 (12.18)	263 (8.75)	
Smoke, %				
Former	2750 (28.02%)	2082 (26.33%)	668 (35.03%)	< 0.0001
Never	5425 (55.28%)	4440 (56.16%)	985 (51.65%)	
Now	1641 (16.71%)	1387 (17.54%)	254 (13.32%)	
Alcohol, %				
Former	1258 (12.82%)	898 (11.35%)	360 (18.88%)	< 0.0001
Mild	3848 (39.20%)	3038 (38.41%)	810 (42.48%)	
Moderate	1678 (17.09%)	1454 (18.38%)	224 (11.75%)	
Heavy	2023 (20.61%)	1791 (22.64%)	232 (12.17%)	
Never	1009 (10.28%)	728 (9.20%)	281 (14.74%)	
BMI, kg/m^2^	31.97 (0.12)	31.76 (0.11)	33.13 (0.22)	< 0.0001
Waist, cm	106.24 (0.30)	105.51 (0.30)	110.35 (0.49)	< 0.0001
PIR	3.07 (0.04)	3.12 (0.05)	2.81 (0.06)	< 0.0001
Glucose, mg/dL	110.23 (0.74)	107.04 (0.66)	128.85 (2.14)	< 0.0001
Insulin, pmol/L	94.17 (1.54)	90.35 (1.56)	116.65 (5.49)	< 0.0001
HbA1c	5.72 (0.01)	5.61 (0.01)	6.31 (0.04)	< 0.0001
eGFR	93.36 (0.51)	97.30 (0.51)	70.86 (0.82)	< 0.0001
Blood urea nitrogen, mmol/L	5.03 (0.04)	4.73 (0.04)	6.73 (0.10)	< 0.0001
Uric acid, μmol/L	337.77 (1.33)	332.28 (1.38)	369.04 (3.03)	< 0.0001
UACR, mg/g	34.59 (3.10)	7.50 (0.11)	186.71 (19.45)	< 0.0001
UALB, μg/mL	35.65 (3.45)	9.45 (0.19)	182.82 (21.73)	< 0.0001
UCR, μmol/L	11176.26 (109.17)	11398.04 (123.26)	9930.46 (198.75)	< 0.0001
Hypertension	4697 (43.34)	3239 (37.94)	1458 (73.21)	< 0.0001
DM	2240 (17.70)	1333 (13.35)	907 (42.89)	< 0.0001
Theaflavin mg/day	1.64 (0.08)	1.68 (0.09)	1.42 (0.12)	0.04
Isorhamnetin mg/day	0.86 (0.02)	0.88 (0.02)	0.69 (0.03)	< 0.001
Luteolin mg/day	0.70 (0.02)	0.77 (0.02)	0.66 (0.03)	0.002
Kaempferol mg/day	4.68 (0.10)	4.79 (0.11)	4.06 (0.20)	0.001
Total flavonoids, mg/day	7.88 (0.18)	8.05 (0.20)	6.91 (0.30)	< 0.0001

### 3.2 Correlation Between Flavanols Intake and Kidney Damage in Hypertensive Patients

To examine the association between flavonoid intake and renal function, we utilized multiple regression models. As shown in Table 2, the overall regression analysis indicated that higher total flavonoid intake was associated with a reduced risk of renal impairment (*p* = 0.02). In Model 3, after adjusting for all relevant covariates, we observed that each 5 mg increase in flavonoid intake was associated with a 4% reduction in the risk of renal impairment (OR = 0.96, 95% CI: 0.92–0.99, *p* = 0.02). In the crude model (Model 1), all four flavonoids were associated with a reduced risk of renal impairment (Table 2). In Model 2, after further adjusting for gender, age, and ethnicity, increased intake of isorhamnetin, luteolin, and kaempferol was associated with a reduced risk of renal impairment (*p* < 0.05). In Model 3, with full adjustment for all variables, only kaempferol intake remained significantly related to renal function decline. Specifically, for every 5 mg increase in kaempferol intake, the risk of renal impairment decreased by 7% (OR = 0.93, 95% CI: 0.88–0.99, *p* = 0.02), with this protective effect identified as an independent factor.

**TABLE 2:** Association of flavonoids intake dosage and kidney damage prevalence.

	Model 1		Model 2		Model 3	
	OR (95% CI)	*p*	OR (95% CI)	*p*	OR (95% CI)	*p*
Total flavonoids	0.94 (0.91, 0.97)	< 0.0001	0.96 (0.93, 0.99)	0.02	0.96 (0.92, 0.99)	0.02
Theaflavin	0.94 (0.89, 0.99)	0.03	0.98 (0.90, 1.05)	0.57	0.96 (0.89, 1.04)	0.35
Isorhamnetin	0.66 (0.51, 0.83)	< 0.001	0.69 (0.54, 0.89)	0.005	0.70 (0.54, 1.01)	0.06
Luteolin	0.62 (0.45, 0.84)	0.002	0.66 (0.46, 0.92)	0.02	0.70 (0.49, 1.01)	0.06
Kaempferol	0.88 (0.83, 0.93)	< 0.0001	0.93 (0.88, 0.98)	0.01	0.93 (0.88, 0.99)	0.02

*Note:* Model 1: No adjusted. Model 2: Adjusted for covariates including gender, age, and ethnicity. Model 3: Model 2 plus variables including smoking status, alcohol intake, PIR, BMI, hypertension, and diabetes mellitus. The dosage of flavonoids intake and subtype intake changed by 5 mg/day.

### 3.3 Effect of Dietary Kaempferol on Kidney Damage and Survival Probability in Overweight and Obese Patients

**FIGURE 1:**
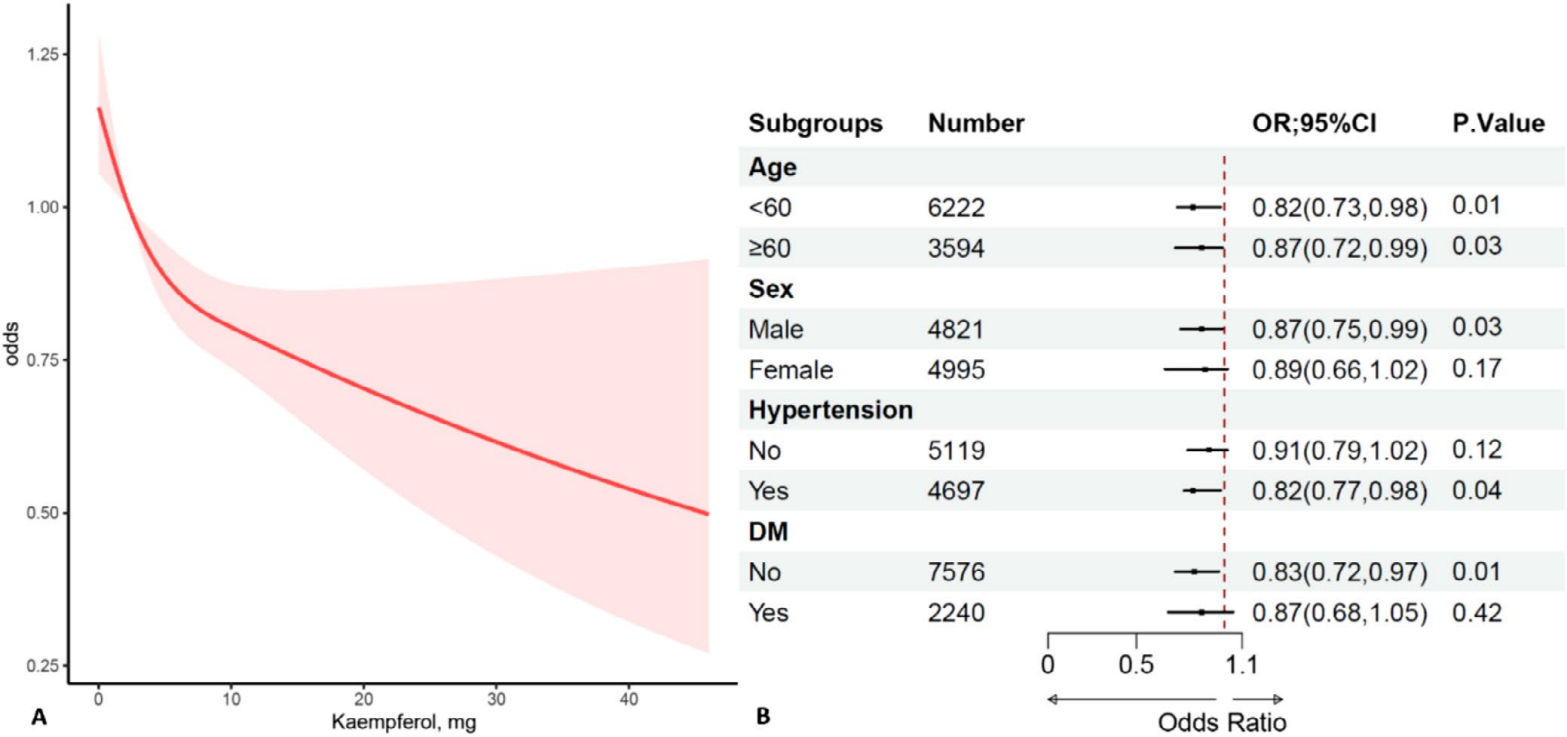
The RCS and subgroup analysis between kaempferol and kidney damage. (A) Indicates that there is an approximately linear negative correlation between kaempferol intake and kidney injury. (B) Shows that in the analysis of different populations except for females, the diabetic population, and the non‐hypertensive population, kaempferol intake is a protective factor for kidney injury.

To further validate the stability of kaempferol's effect on renal function, we categorized kaempferol intake into tertiles (T1, T2, T3). The average daily intake of kaempferol for the three groups was 0.59 mg (3.01 ± 0.01), 2.44 mg (8.04 ± 0.02), and 9.73 mg (20.38 ± 0.16), respectively (Table 3). After incorporating the tertile‐based kaempferol intake into the regression models, the T3 group consistently showed a reduced risk of renal impairment compared to the T1 group across all models (Model3: OR = 0.82, 95% CI: 0.71–0.94, *p* = 0.02). RCS analysis showed an approximately linear negative correlation between kaempferol intake and the risk of kidney damage (Figure 1A). To further validate the effect of kaempferol intake across different populations, we conducted a subgroup analysis and presented the results in a forest plot (Figure 1B). The analysis revealed that dietary kaempferol had a significant protective effect in individuals with hypertension, diabetes, and among the elderly (*p* < 0.05).

**TABLE 3:** Association of kaempferol intake dosage and kidney damage prevalence.

	Kaempferol (mg/day)	Model 1		Model 2		Model 3	
		OR (95% CI)	*p*	OR (95% CI)	*p*	OR (95% CI)	*p*
Kaempferol	—	0.88 (0.83, 0.93)	< 0.0001	0.93 (0.88, 0.98)	0.01	0.93 (0.88, 0.99)	0.02
Kaempferol group							
T1	0.59 (0.01)	Ref		Ref		Ref	
T2	2.44 (0.02)	0.93 (0.83, 1.05)	0.27	0.93 (0.82, 1.06)	0.30	0.97 (0.85, 1.10)	0.61
T3	9.73 (0.16)	0.72 (0.64, 0.82)	< 0.0001	0.78 (0.68, 0.89)	< 0.001	0.82 (0.71, 0.94)	0.01

*Note:* Model 1: No adjusted. Model 2: Adjusted for covariates including gender, age, and ethnicity. Model 3: Model 2 plus variables including smoking status, alcohol intake, PIR, BMI, hypertension, and diabetes mellitus. Taking the T1 group as the reference value.

A median follow‐up time of 117 months was obtained. Kaplan–Meier analysis showed significantly higher survival probability in the high kaempferol intake group (*p* < 0.001; Figure S2). Multivariate Cox regression analysis further revealed that for each 5 mg increase in kaempferol intake, the long‐term mortality rate among overweight or obese individuals decreased by 7% (HR = 0.93, 95% CI: 0.85–0.98, *p* = 0.01) (Table 4). In Model 3, the mortality rate in the highest intake group (T3) was 0.7 (95% CI: 0.6–0.83) times that of the lowest intake group (T1), indicating that kaempferol intake is beneficial for the long‐term prognosis of overweight or obese individuals. RCS and subgroup analyses supported a linear association and consistent survival benefit across key subgroups (Figures S3A and S3B).

**TABLE 4:** Association of kaempferol intake dosage and long term prognosis among overweight and obese individuals.

	Model 1		Model 2		Model 3	
	HR (95% CI)	*p*	HR (95% CI)	*p*	HR (95% CI)	*p*
Kaempferol	0.87 (0.81, 0.95)	0.001	0.91 (0.84, 0.97)	0.01	0.93 (0.85, 0.98)	0.01
Kaempferol group						
T1	Ref		Ref		Ref	
T2	0.83 (0.69, 0.94)	0.01	0.81 (0.68, 0.96)	0.01	0.81 (0.69, 0.95)	0.02
T3	0.59 (0.49, 0.70)	< 0.001	0.70 (0.59, 0.84)	< 0.001	0.70 (0.60, 0.83)	< 0.001

*Note:* Model 1: No adjusted. Model 2: Adjusted for covariates including gender, age, and ethnicity. Model 3: Model 2 plus variables including smoking status, alcohol intake, PIR, BMI, hypertension, and diabetes mellitus. The dosage of Kaempferol intake changed by 5 mg/day. Taking the T1 group as the reference value.

### 3.4 Sensitivity Analysis

In the sensitivity analysis, we employed two approaches. First, we reclassified kaempferol intake into two groups based on the median: a low intake group and a high intake group. Second, we reassessed the impact of kaempferol on renal function and long‐term prognosis across different follow‐up periods. Even after fully adjusting for covariates, the high intake group among the overweight or obese population continued to demonstrate a protective effect on renal function and favorable long‐term prognosis across all follow‐up cycles (Table S1).

## 4 Discussion

Obesity and overweight are crucial risk factors for kidney injury, and this kind of renal function impairment may be associated with an increased risk of various chronic diseases (Ye et al. 2022). These health issues require long‐term and multifaceted management strategies (Chang et al. 2019). Although lifestyle adjustments are recommended as the primary preventive measure, specific nutritional supplements, such as kaempferol, may offer additional benefits for improving renal function and long‐term health outcomes. Kaempferol is a flavonoid compound widely present in nature and has attracted attention due to its diverse biological activities and potential health benefits (Alkandahri et al. 2024). Currently, most of the results regarding the protective effect of kaempferol on the kidneys were from animal experiments (Alshehri 2023; Wang et al. 2020; Luo et al. 2021; Varela‐Rodriguez et al. 2024), and there were no relevant studies based on humans yet. Making use of data from the NHANES database, this research was aimed at exploring the influence of dietary kaempferol on kidney impairment and the long‐term prognosis in obese and overweight individuals. In this study, we primarily analyzed four types of flavonoids. Overall, an increase in flavonoid intake was associated with a reduction in the prevalence of kidney injury. In the subtype analysis, after adjusting for multiple covariates, it was found that increased kaempferol intake significantly reduced the risk of renal function decline, with this protective effect being independent of blood glucose and blood pressure levels. Specifically, for every 5 mg increase in kaempferol intake, the prevalence of kidney injury decreased by 7%. To further investigate the relationship between kaempferol intake and the risk of kidney injury, we categorized participants into three intake groups. Compared to the low‐intake group, the high‐intake group showed a significant reduction in the prevalence of kidney injury. Additionally, a follow‐up period of up to 117 months was conducted to assess the long‐term prognostic impact of kaempferol on the obese and overweight population. Multivariate Cox regression analysis revealed that for every 5 mg/day increase in kaempferol intake, mortality in the obese and overweight population was reduced by 8%, an effect independent of blood glucose and blood pressure. In the three‐group analysis, the highest kaempferol intake group had a 30% lower risk of mortality (HR = 0.7) compared to the low‐intake group. Further subgroup analyses demonstrated that the protective effect of kaempferol on kidney function and the improvement in long‐term prognosis were consistent across various subpopulations. However, in the diabetic population, kaempferol intake did not show a significant impact on reducing kidney injury risk. In contrast, in individuals with both diabetes and hypertension, dietary kaempferol was associated with a significant improvement in prognosis.

Our study identified that higher kaempferol intake was independently associated with improved renal function and better long‐term prognosis in overweight or obese adults. The renoprotective effect of kaempferol observed in our study may stem from its intervention in the core mechanisms of obesity‐related kidney injury. Obesity damages the kidneys through multiple pathways: direct mechanisms include glomerular hyperfiltration and lipotoxicity‐induced structural damage (Ahmed et al. 2025; Brennan et al. 2021), while indirect mechanisms involve chronic low‐grade inflammation, oxidative stress, and RAAS overactivation driven by dysfunctional adipose tissue (Dousdampanis et al. 2024; Verde et al. 2023). These processes collectively lead to renal fibrosis and functional decline. The antioxidant and anti‐inflammatory properties of kaempferol are precisely positioned to counteract these key pathological events. Kaempferol is a polyphenolic compound widely found in the plant kingdom, commonly present in vegetables, legumes, fruits, and other plant‐based foods. It offers various potential health benefits, particularly demonstrating a unique mechanism of action and promise in kidney protection. In terms of kidney protection mechanisms, kaempferol exerts multiple beneficial effects. It effectively reduces the production of reactive oxygen species (ROS) (He et al. 2017) and enhances the activity of antioxidant enzymes, such as superoxide dismutase and catalase (Molaei et al. 2021), thereby increasing the levels of nuclear factor erythroid 2‐related factor 2 (Nrf2) and heme oxygenase 1 (HO‐1) (Luo et al. 2021). These actions help mitigate kidney damage caused by oxidative stress. Additionally, kaempferol decreases the release of inflammatory cytokines, such as interleukin‐12 (IL‐12) (Li et al. 2021) and tumor necrosis factor‐α (TNF‐α) (Malik et al. 2017), and inhibits the activity of nuclear factor κB (NF‐κB) (Wang et al. 2020), effectively suppressing the inflammatory response and alleviating inflammation‐induced kidney damage. In terms of regulating apoptosis, kaempferol inhibits the activation of mitogen‐activated protein kinase (MAPK) (Kim et al. 2016) and NF‐κB, reduces levels of protein 53 (p53), blocks apoptosis‐related pathways, and protects kidney cells from apoptosis (Marfe et al. 2009). Furthermore, kaempferol activates the SIRT1 signaling pathway, inhibits fibrosis mediated by transforming growth factor‐β1 (TGF‐β1) (Liu et al. 2021), and reduces kidney fibrosis (Zhu et al. 2011). In diabetic nephropathy, kaempferol improves mitochondrial function, reduces oxidative stress, inflammation, and apoptosis caused by mitochondrial dysfunction, and regulates the expression of autophagy proteins to maintain mitochondrial health (Alkandahri et al. 2024). Clinically, kaempferol is abundant in various plant‐based foods such as broccoli, spinach, and onions, and can be easily consumed as part of a regular diet. However, one major challenge in the clinical application of kaempferol is its low bioavailability (Dabeek and Marra 2019). Emerging technologies, such as nanotechnology, offer promising solutions to this issue (Alkandahri et al. 2024). By enhancing kaempferol's bioavailability, nanotechnology could significantly boost its efficacy, providing a promising avenue for developing kaempferol as a kidney protectant in the future. This development is anticipated to play a more prominent role in the prevention and treatment of kidney diseases. Furthermore, the results of the survival analysis indicated that supplementing with kaempferol was conducive to enhancing the survival rate of obese or overweight patients and improving the prognosis. All the aforementioned improvements were independent of blood glucose and blood pressure levels, suggesting that recommending dietary kaempferol as an adjunctive treatment method for obese or overweight patients.

Our study has several limitations. Firstly, due to the cross‐sectional study design, we could only explore the association between kaempferol intake and the prevalence of kidney injury but not its incidence. Secondly, the NHANES database does not provide information on the duration of flavonoid use, including kaempferol, which limits the direct applicability of our findings to clinical practice. Thirdly, residual confounding from unmeasured factors, such as overall diet quality or intake of other bioactive compounds, cannot be fully ruled out. Additionally, the survival follow‐up period was relatively short, with participants tracked for approximately 10 years. Over time, the impact of dietary kaempferol on long‐term prognosis may become more pronounced, but this requires further verification through future updates.

Despite these limitations, our study has notable strengths. This is the first study to systematically investigate the effects of dietary kaempferol on kidney function and long‐term prognosis in overweight and obese individuals. While previous research demonstrated the protective effects of kaempferol on the kidneys in laboratory and animal models, our study addresses the growing burden of obesity‐related CKD in a human population, filling a critical gap in the literature. Moreover, our comprehensive assessment of renal biomarkers and long‐term outcomes offers novel insights into the therapeutic potential of kaempferol. Given the rising global prevalence of obesity and its strong association with CKD, our findings provide valuable evidence supporting kaempferol as a clinically viable, natural adjunct therapy for improving kidney health in this population.

## 5 Conclusion

In overweight and obese populations, higher dietary kaempferol intake is associated with a lower prevalence of kidney injury and improved prognosis. These findings support the potential benefit of dietary kaempferol and warrant future randomized controlled trials to confirm causality before clinical recommendations can be made and elucidate the biological mechanisms of kaempferol in kidney protection.

## Author Contributions

**Lin Shi:** conceptualization (equal), data curation (equal), formal analysis (equal), funding acquisition (equal), investigation (equal), methodology (equal), project administration (equal), resources (equal), software (equal), supervision (equal), validation (equal), visualization (equal), writing – original draft (equal), writing – review and editing (equal). **Yiquan Sang:** conceptualization (equal), data curation (equal), formal analysis (equal), funding acquisition (equal), investigation (equal), methodology (equal), project administration (equal), resources (equal), software (equal), supervision (equal), validation (equal), visualization (equal), writing – original draft (equal), writing – review and editing (equal).

## Funding

Affiliated Hospital Development Fund Project of Xuzhou Medical University (XYFY202203). The social development project of the Xuzhou Municipal Science and Technology Bureau (KC23183).

## Ethics Statement

The studies involving human participants were reviewed and approved by The National Center for Health Statistics Research Ethics Review Board. The patients/participants provided their written informed consent to participate in this study. All methods were carried out in accordance with relevant guidelines and regulations (Declaration of Helsinki).

## Consent

The authors have nothing to report.

## Conflicts of Interest

The authors declare no conflicts of interest.

## Supporting information


**Data S1:** fsn371393‐sup‐0001‐Supinfo.docx.

## Data Availability

Publicly available datasets were analyzed in this study. This data can be found here: https://www.cdc.gov/nchs/nhanes.
