# Supplementary material for: Beneficial Impact of Kaempferol on Kidney Function and Long‐Term Prognosis in Overweight or Obese Adults
Source: Food Sci Nutr. 2026 Jan 27;14(1):e71393. doi: 10.1002/fsn3.71393 (PMC12836288; doi:10.1002/fsn3.71393)
Supplement: Supplementary file 1 — Data S1: fsn371393‐sup‐0001‐Supinfo.docx. [file FSN3-14-e71393-s001.docx]

Supplementary material

Table S1 Association between Kaempferol and the prevalence of kidney damage and long term prognosis in different cycles

|  | 2007-2008 | | 2009-2010 | | 2017-2018 | |
| --- | --- | --- | --- | --- | --- | --- |
|  | OR (95% CI) | P value | OR (95% CI) | P value | OR (95% CI) | P value |
| Kaempferol group |  |  |  |  |  |  |
| T1 | Ref |  | Ref |  | Ref |  |
| T2 | 0.87(0.69,0.98) | 0.03 | 0.81(0.66,0.99) | 0.04 | 0.85(0.65,0.99) | 0.04 |
|  | HR (95% CI) | P value | HR (95% CI) | P value | HR (95% CI) | P value |
| Kaempferol group |  |  |  |  |  |  |
| T1 | Ref |  | Ref |  | Ref |  |
| T2 | 0.71(0.59,0.85) | <0.001 | 0.78(0.63,0.98) | 0.01 | 0.77(0.35,0.99) | 0.05 |

Note: After grouping kaempferol by the median, we conducted logistic and Cox multivariate regression analyses respectively for different cycles. The adjusted variables were the ones in model 3, and the presented results were also those of model 3.


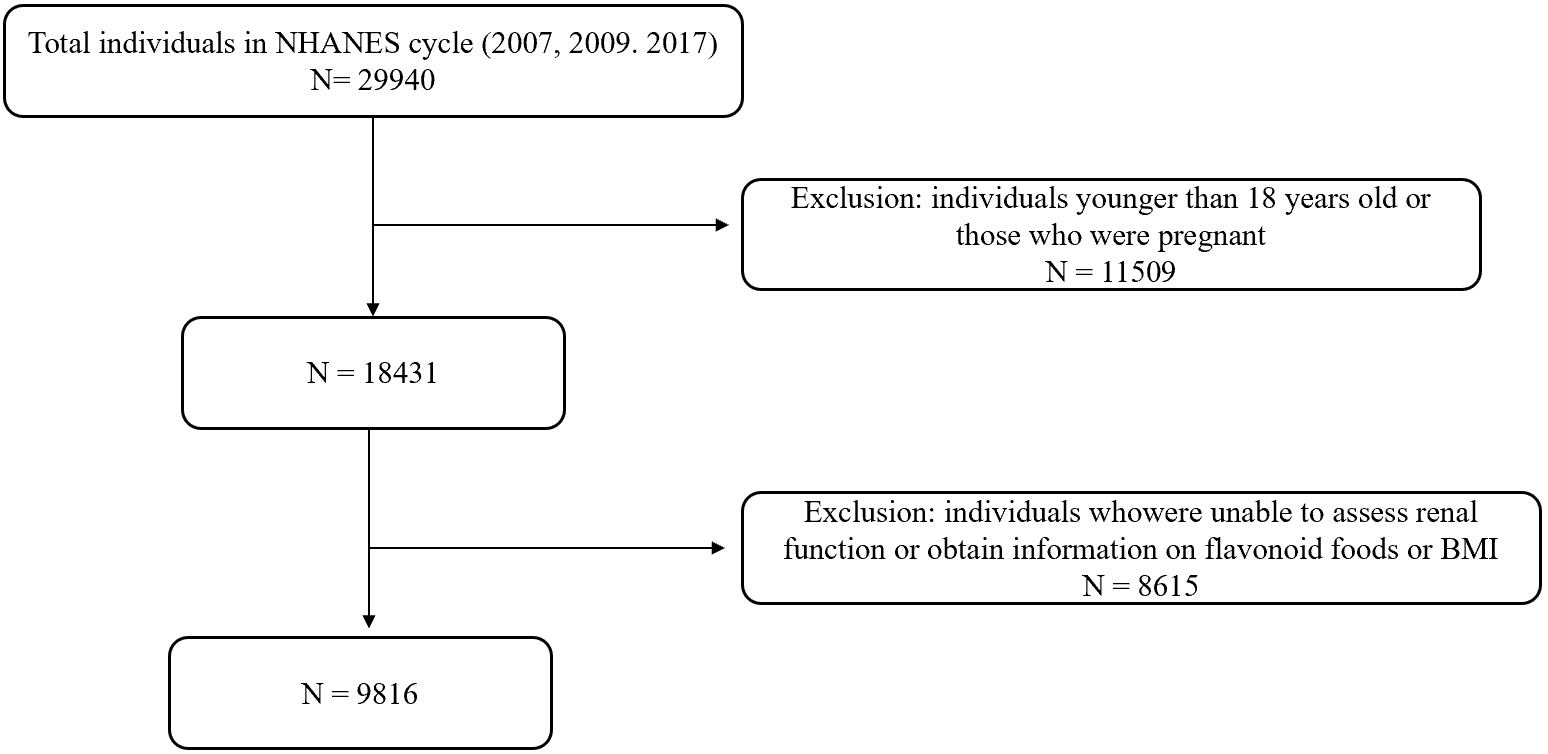


Figure S1: Flowchart for participant selection in this study.


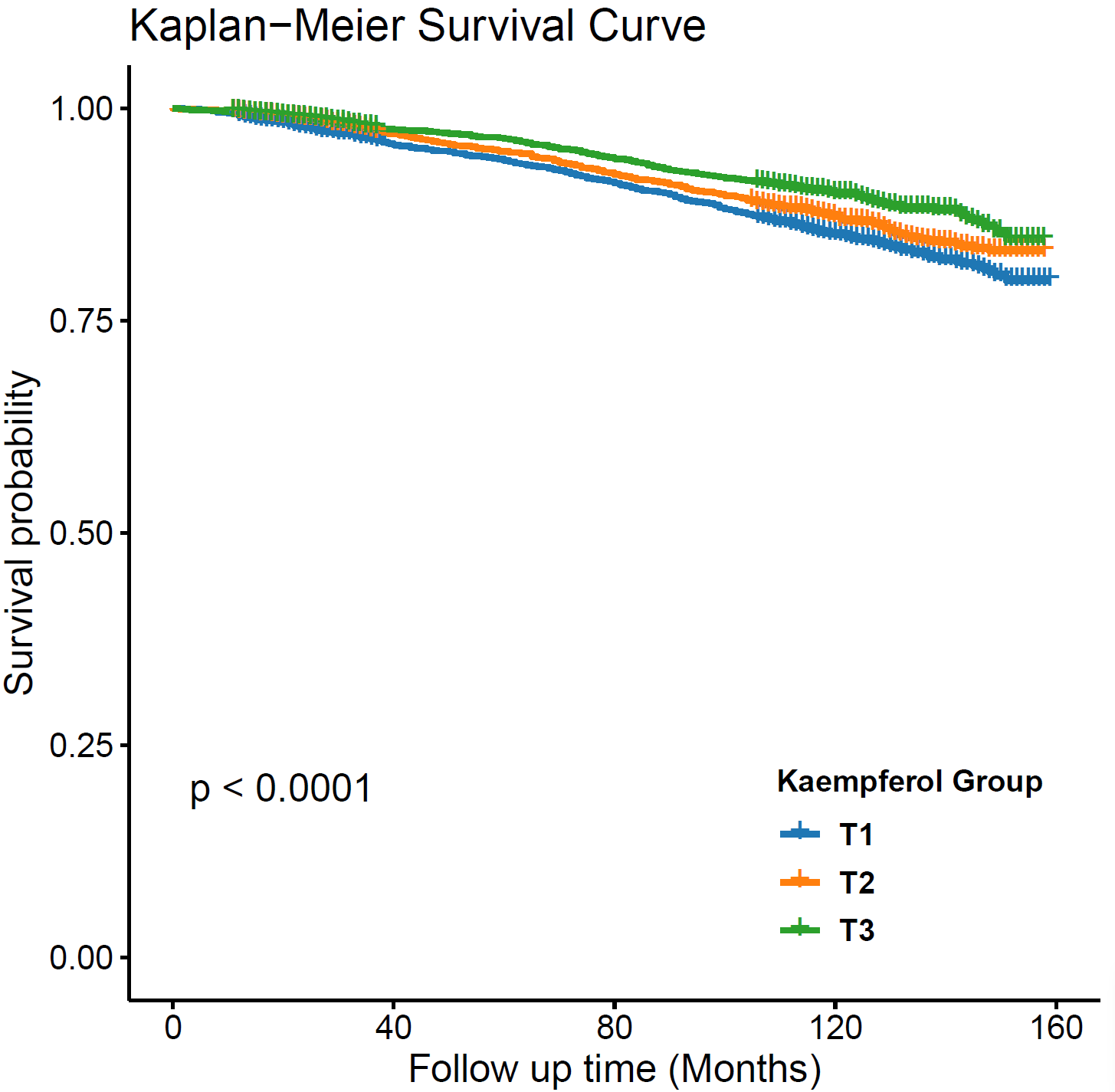


Figure S2: The Kaplan–Meier survival curve regarding the effect of kaempferol supplementation on the survival probability among obese and overweight individuals. The Kaplan–Meier survival curve was employed to illustrate the connections between kaempferol supplementation and the survival probability in obese and overweight individuals.


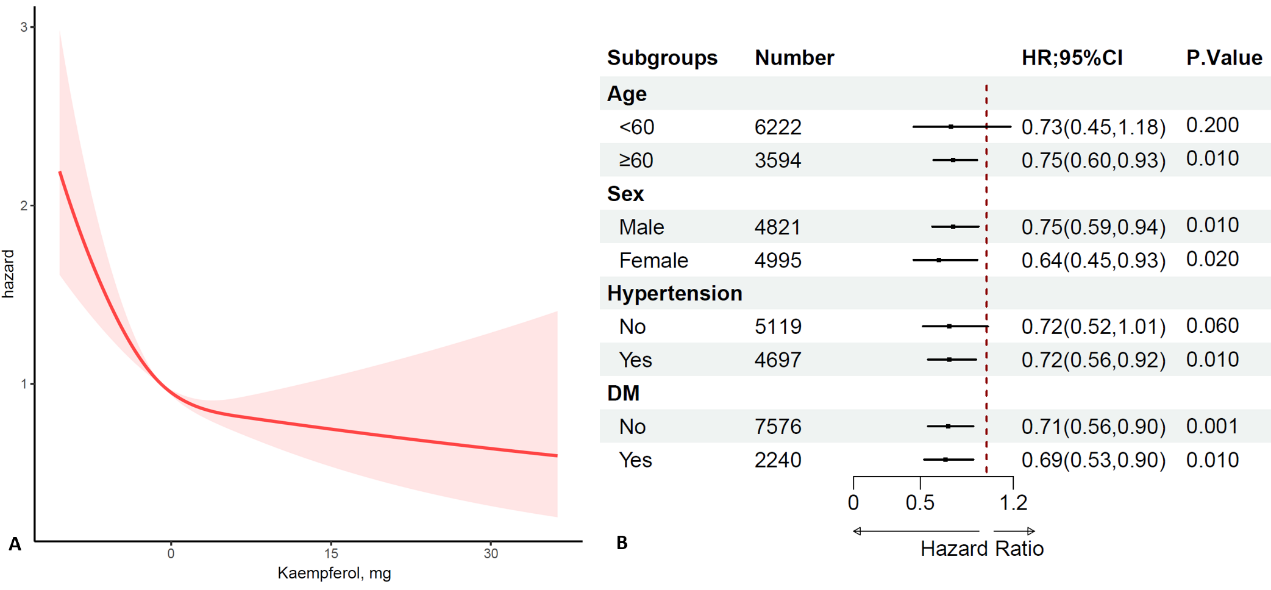


Figure S3: The RCS and subgroup analysis between kaempferol and long term prognosis. Figure 4A suggests that an increase in kaempferol intake may reduce the mortality rate. Figure 4B suggests that kaempferol has a good effect on improving prognosis in different subgroup analyses.
